# DRINKING WATER QUALITY, DYSLIPIDEMIA, AND COGNITIVE FUNCTION FOR OLDER ADULTS IN CHINA

**DOI:** 10.1093/geroni/igac059.328

**Published:** 2022-12-20

**Authors:** Xi Pan, Ye Luo, Dandan Zhao, Lingling Zhang

**Affiliations:** Texas State University, San Marcos, Texas, United States; Clemson University, Clemson, South Carolina, United States; Clemson University, Clemson, South Carolina, United States; University of Massachusetts Boston, Boston, Massachusetts, United States

## Abstract

The current study aimed to examine the associations between drinking water quality and cognitive function and to identify the direct and indirect effects of drinking water quality and dyslipidemia on cognitive function among older adults in China. Data for the study were selected from China Health and Retirement Longitudinal Study (CHARLS, 2015) and 4,951 respondents aged 60 and above were included. Dyslipidemia was measured by self-reported dyslipidemia diagnosis and lipid panel. Three composite measures of cognitive function included mental status, episodic memory, and global cognition. Mixed effects models were conducted to assess the associations between drinking water quality or dyslipidemia and cognitive function. The mediation effects of dyslipidemia were examined by path analyses. Results showed that exposure to high quality drinking water was significantly associated with higher scores in mental status, episodic memory, and global cognition (B = 0.31, p< 0.001 for mental status; B= 0.22, p< 0.05 for episodic memory; B = 0.53, p < 0.01 for global cognition). Elevated blood triglycerides was associated with higher scores in mental status and global cognition (B = 0.21, p< 0.05 for mental status; B = 0.32, p< 0.05 for global cognition). Self-reported dyslipidemia diagnosis was a suppressor, which increased the magnitude of the direct effect of drinking water quality on mental status and global cognition. Findings of the study suggest that improving drinking water quality could be a potential public health effort to delay the onset of cognitive impairment and prevent the dementia pandemic in older people.

